# Viral metagenomic analysis of fecal samples reveals an enteric virome signature in irritable bowel syndrome

**DOI:** 10.1186/s12866-020-01817-4

**Published:** 2020-05-19

**Authors:** Mina Hojat Ansari, Mehregan Ebrahimi, Mohammad Reza Fattahi, Michael G. Gardner, Ali Reza Safarpour, Mohammad Ali Faghihi, Kamran Bagheri Lankarani

**Affiliations:** 1 Gastroenterohepatology Research Center, Shiraz University of Medical Sciences, Shiraz, Iran; 2 Health Policy Research Center, Institute of Health, Shiraz University of Medical Sciences, Shiraz, Iran; 3 Department of Biology, Shiraz University, Shiraz, Fars Province Iran; 4 College of Science and Engineering, Flinders University, Adelaide, South Australia Australia; 5 Evolutionary Biology Unit, South Australian Museum, Adelaide, Australia; 6 Persian Bayan Gene Research and Training Center, Dr. Faghihi’s Medical Genetics Center, Shiraz, Iran; 7 Center for Therapeutic Innovation, Department of Psychiatry and Behavioral Sciences, University of Miami Miller School of Medicine, Miami, FL USA

**Keywords:** Irritable bowel syndrome, Microbiota, Enteric virome, Metagenomics analysis, Bacteriophages, Eukaryotic viruses

## Abstract

**Background:**

Changes in the enteric microbiota have been suggested to contribute to gastrointestinal diseases, including irritable bowel syndrome. Most of the published work is on bacterial dysbiosis with meager data on the role of the virome in irritable bowel syndrome and other gastrointestinal diseases. In the current study, we therefore aimed to investigate the viral community composition of the gut and test for potential dysbiosis linked to irritable bowel syndrome.

**Results:**

A metagenomics analysis on fecal samples of 50 individuals — 30 of whom met the Rome IV criteria for IBS and 20 healthy controls— was conducted. There was a noticeable alteration in viral taxa observed in association with irritable bowel syndrome when compared to healthy individuals — where some eukaryotic viral taxa noticeably prevail over others. We observed a significant decrease in the diversity and abundance of enteric virome particularly in eukaryotic viruses of Megavirales in patients with irritable bowel syndrome.

**Conclusions:**

These findings shed light on a new hypothesis that the alteration of the viral taxa contributes to the pathogenesis of irritable bowel syndrome and related symptoms, and therefore, pave the way for developing a new diagnostic biomarker or anti-viral drugs for the treatment of irritable bowel syndrome.

## Background

Irritable bowel syndrome (IBS) is a functional bowel disease which is characterized by abdominal pain, bloating and irregular defecation [1]. The etiology of IBS is undeniably multifactorial — with genetic and environment both having important roles. One of the environmental factors that are broadly attributed to the pathogenesis of IBS is the composition of the microbiota that resides in the intestine [2–4]. The human microbiome consists of nearly 100 trillion cells, which is equal or even exceeds the number of somatic and germ cells comprising the human body [5, 6]. These cells are mainly bacteria, however, they also include viruses, archaea and microeukaryotes [7, 8]. Excluding the bacteria, studies on other microorganisms — particularly viruses and their association with human health — are limited [9, 10].

In general, the composition of the viral community of the microbiota is difficult to analyze because the genetic material of the viruses can have both DNA and RNA. In addition, a conserved sequence platform that can be used for compositional analysis is missing [11]. However, newly developed sequencing technology has contributed to the study of enteric human virome and highlighting their potential association in health and disease [12–14].

Elucidated from studies using new technologies and the existing literature, the number of viruses resides in the human intestine are estimated to be up to 10^9^ per gram of feces [15], comprising mainly of bacteriophages (prokaryotic-infecting viruses), and to a lesser extent plant, amoebae, human, and other animal infecting viruses [12, 16–18]. The human virome acquired mostly postnatally that influenced by a combination of dietary, maternal and environmental sources [19, 20]. During the first week after birth, the number of gut viruses estimated at 10^8^ g in feces but infant virome develops, diversifies and reaches its peak by adulthood [21]. The enteric viruses interact with other viruses, adenovirus and bacteria inside the body and our cells, therefore, may have a direct impact on both our health and disease [10]. Notably, a unique increase of bacteriophages Caudovirales has been observed in association to decreased bacterial diversity in patients with Crohn’s disease, emphasizing that bacterial dysbiosis and intestinal inflammation could attribute to an unbalance virome composition [22]. Alternatively, epidemiological studies in animal models showed that viruses could have beneficial effects on health [23]. For instance, it has been found that Herpesviruses could activate Natural Killer cells and increase resistance to tumor grafts [24–26].

Eukaryotic targeting viruses are less abundant compared to bacteriophages but are able to transfer their genetic information directly to host cells and therefore effectively stimulate host immune responses [27]. Evidence from several human and animal studies showed that eukaryotic viruses could hold a persistent immune response and so may increase the susceptibility of the host to disease [28]. Yet, recent studies showed that some eukaryotic viruses, which are considered pathogenic, also frequently reside in the healthy human intestine, but without any causing symptoms [29, 30]. Therefore, eukaryotic viruses, even from some pathogenic groups such as *Parvoviridae*, *Anelloviridae*, *Picobirnaviridae*, *Circoviridae*, and *Reoviridae*, often are part of the enteric virome of healthy humans [29], and so participate in the physiology of intestine. Accordingly, it is now clearly understood that enteric eukaryotic viruses are primary modulators of intestinal homeostasis and immune responses because they are constantly must be controlled by local defense mechanisms for preventing the development of intestinal pathology.

Studies used pathogenic viruses indicated that specific molecules from host such as TLR, RIG-I, MDA5 and several pathways are likely to detect the presence of viruses and modulate the immune response based on the local requirements [28, 31–34]. However, it is not yet understood which viral sensing and signaling pathways are important for adjusting the immune responses to control the abundance and composition and so the effect of the enteric viruses.

However, to our knowledge, the gut viral composition has only been assessed in association to colorectal cancer and inflammatory bowel disease (IBD) [35–37] with limited data available from IBS patients. A previous study evaluated gut bacterial community composition in both IBD and IBS patients and proposed the possibility of overlapping pathophysiology between IBS and IBD — including the processes of inflammatory that lead to a reduction of diversity in microbiota [4].

Here we analyzed the viral composition of fecal samples from healthy controls (HC) and patients with early-diagnosed IBS using the metagenomics analysis. We aimed to characterize the viral community composition of the gut and test for potential dysbiosis in association with IBS.

## Results

### Study population and taxonomic assignment

Out of a total of 50 individuals, 42 (25 IBS patients and 17 HC) passed the filtering step based on the number of reads and therefore had sufficient data to be used for the final analysis. The studied population consisted of 26 (61.9%) women and 16 (38.1%) men with an average age of 34 ± 0.06 years old. The IBS patients were subcategorized based on their symptoms and following the Rome IV criteria for IBS. Therefore, four clinically relevant groups were determined consisted of: eight IBS patients with symptoms of predominant constipation (IBS-C), seven with diarrhea-predominant (IBS-D), 10 patients with mixed-bowel symptoms (IBS-M), and 17 HC. Abdominal pain was the most commonly reported symptom followed by abdominal bloating which occurred in 88% of patients with IBS. We also found 16% of patients with IBS rated abdominal bloating as the first most troublesome symptom. The demographic characteristics of the subjects are presented in Additional file 1: Table S1.

We obtained on average 10,564,705 ± 5,498,331 sequences (Number ± standard deviation) from 42 individuals (Additional file 1: Table S2). Quality control and length trimming resulted in an average nearly 1% reduction in the number of sequences to 10,512,984 ± 5,473,216. The majority of sequences obtained in the current study were assigned to bacteria, which were excluded from the current study (data not presented).

### Characterization of the enteric virome from IBS patients and HC

We were able to taxonomically assign an average of 19% of sequences to viral genomes, of which more than 90% were identified as double-strand (ds) DNA viruses. The most abundant viral taxa in both IBS patients and HC were identified as bacteriophages of the Caudovirales order (*Myoviridae*, *Podoviridae*, and *Siphoviridae*), which was followed by eukaryotic viruses from the order Megavirales. The taxonomic assignment of the sequences also indicated many less abundant viral taxa which were classified in Herpesvirales and Ligamenvirales orders (Additional file 2: Fig. S1). Accordingly, the enteric virome of IBS patients predominantly consisted of viral sequences from the families *Myoviridae* (22.7%), *Poxviridae* (13.94%), *Siphoviridae* (12.27%), *Mimiviridae* (10.88%), *Herpesviridae* (4.34%), *Phycodnaviridae* (3.48%), *Baculoviridae* (2.62%), *Marseilleviridae* (2.54%), *Podoviridae* (2.19%), *Pandoraviridae* (1.81%), *Iridoviridae* (1.33%), *Nudiviridae* (0.34%), *Rudiviridae* (0.15%), *Adenoviridae* (0.08%), *Hytrosaviridae* (0.05), *Alloherpesviridae* (0.04), *Nimaviridae* (0.03) and *Polydnaviridae* (0.001).

Viral species from *Myoviridae* (29.42%), *Poxviridae* (13.6%), *Siphoviridae* (11.68%), and *Mimiviridae* (9.65%) also were the highest abundant taxa in HC. This trend then followed with species from *Phycodnaviridae*, *Iridoviridae*, *Podoviridae*, *Marseilleviridae,* and *Baculoviridae*, which showed a relatively low abundance (from 4 to 2%). However, viral species from *Herpesviridae*, *Pandoraviridae*, *Nudiviridae*, *Rudiviridae*, *Adenoviridae*, *Polydnaviridae*, *Hytrosaviridae*, *Alloherpesviridae*, and *Nimaviridae* (with less than 2% relative abundance rate) were the least enriched taxa in healthy individuals (Additional file 2: Fig. S2).

**Fig. 1:**
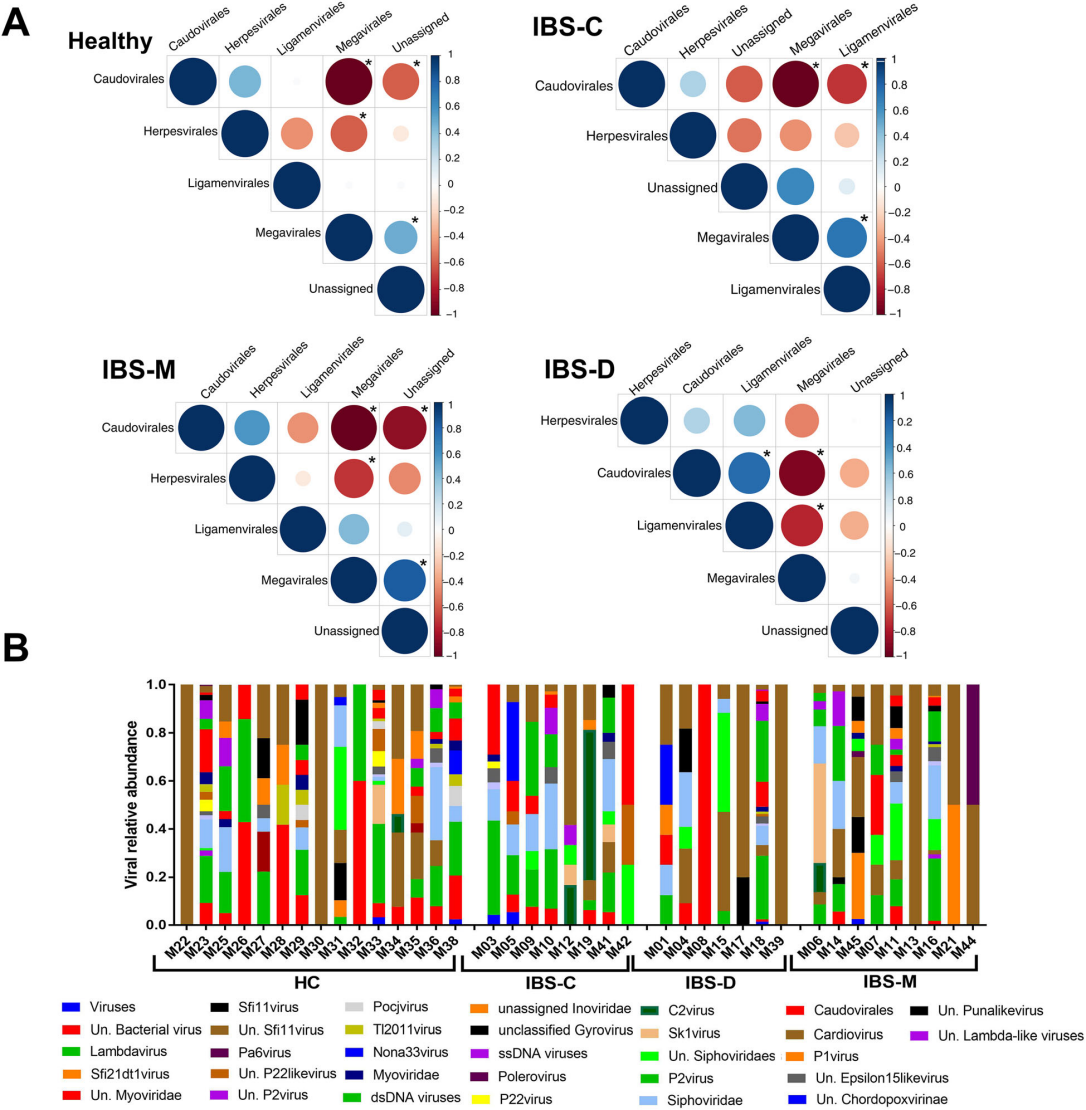
The assigned viral taxa in association with IBS and healthy controls. **a**: Spearman correlation plot of the relative abundance for all the assigned viral order in Healthy, IBS-C, IBS-D, and IBS-M. An asterisk indicates a statistically significant (*p* values < 0.05) for the pairwise comparison. Positive values (Blue circles) corresponding to the positive correlation and negative values (red circles) refer to inverse correlations. The scale of the correlation presented with the size and shading of the circles where darker shades indicate higher correlation compared with lighter shades. **b**: Plots of the relative abundance of the viral taxa assigned to the assembled contigs. The data presented based on the viral genus level

The result of relative abundance analysis shown an inverse correlation between the Caudovirales and Megavirales (Fig. 1A, Additional file 1: Table S3). Interestingly, a positive correlation between Megavirales and Ligamenvirales and an inverse correlation between Caudovirales and Ligamenvirales was detected in IBS-C when tested for each IBS subtype separately (Fig. 1A, Additional file 1: Table S3). But an inverse correlation between Megavirales and Ligamenvirales and a positive correlation between Caudovirales and Ligamenvirales observed in IBS-D group. Furthermore, an inverse correlation between Megavirales and Herpesvirale*s* was found in HC and IBS-M groups separately (Fig. 1A, Additional file 1: Table S3).

Taxonomic assignment of de-novo assembled contigs of more than 300 bp in length following comparison to the viral genomes indicated bacteriophages of the order Caudovirales had the highest relative abundance (Fig. 1B). In addition, we mapped our trimmed sequences to *crAssphage* genome, which has been discovered by [38], and detected contamination in just 7.14% of our samples. Interestingly, when we mapped the assembled sequences (longer than 300 bp) back to the *crAssphage* genome, we discovered *crAssphage* in 14.3% of our samples (Additional file 1: Table S2).

### Viral α and β diversity associated with IBS

**Fig. 2:**
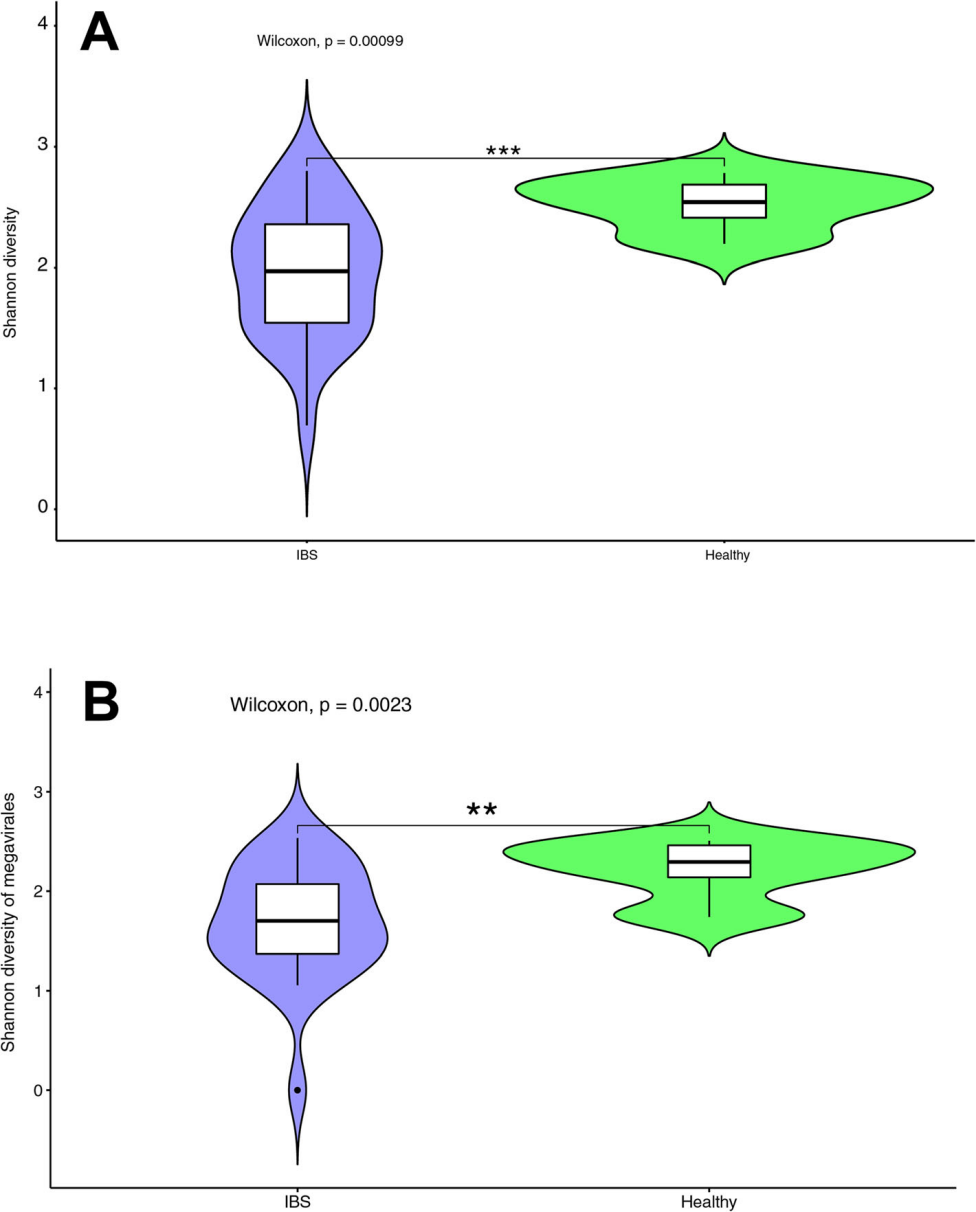
The diversity of the viral taxa assigned to the identified sequences in the current study. Wilcoxon sum test was used to test for statically significant variation. **a**: Total viral diversity differences between healthy controls and patients with IBS. **b**: The diversity assessment of Megavirales based on the individual sequences between healthy individuals and IBS patients

**Fig. 3:**
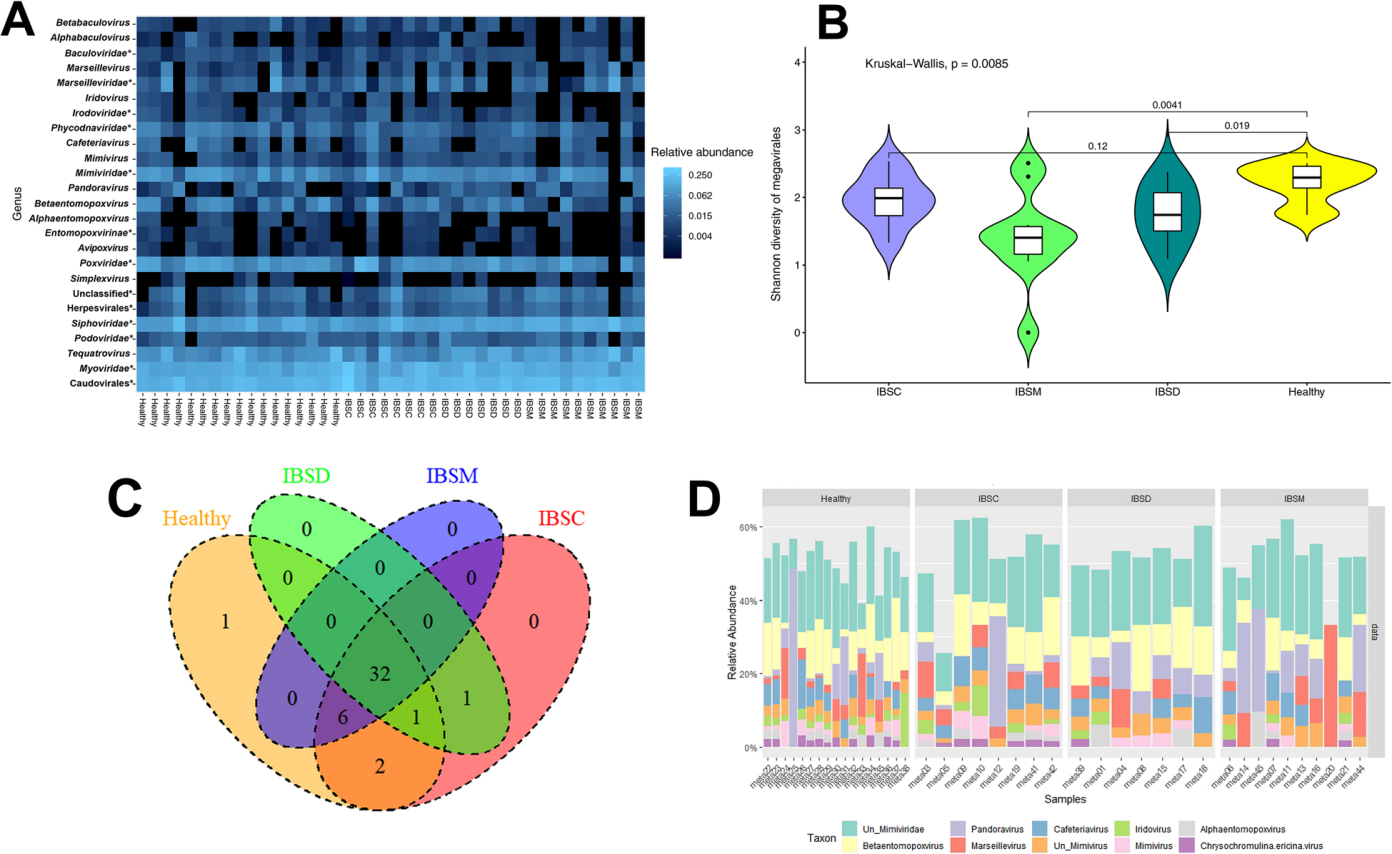
Megavirales dysbiosis contributes to the IBS and the associated symptoms. **a**: Heat map cauterization based on the presence and absence of the assigned sequences to Megavirales. **b**: The diversity of Assigned Megavirales sequences between healthy controls and different IBS subtypes. The median and interquartile range were presented with the bars. The significance of the observed variation was statistically determined using the Wilcoxon sum test. **c**: Venn diagram of the Megavirales taxa in healthy control and different IBS subtypes. **d**: The relative abundance of the 10 most abundant Megavirales genus in the different subtypes of IBS and healthy control

Total viral diversity differed significantly between patients with IBS and HC (*p* = 9.9 × 10^− 4^ Wilcoxon test, Fig. 2A). Those suffering from IBS had significantly fewer viral species (Fig. 2A), especially members of the Megavirales, than HC (*p* = 2.3 × 10^− 3^ Wilcoxon test, Fig. 2B). Further, we observed significant variation in the diversity of the Megavirales taxa among IBS subgroups, where the diversity of Megavirales in patients with IBS-D and IBS-M were significantly varied from the HC (p _IBS-D_ = 0.019, p _IBS-M_ = 0.004 Wilcoxon test, Fig. 3A, B). The results showed HC harbored one unique Megavirales species, but no unique species were identified for any of the IBS subtypes (Fig. 3C). However, two species from Megavirales were shared in just between HC and IBS-C patients, and no species found to be unique between HC and IBS-D or IBS-M patients (Fig. 3C, Additional file 1: Table S4). Patients with IBS-M shared in six unique Megavirales species with HC and IBS-C subjects, where just one species identified to be shared between IBS-D, HC, and IBS-C samples. Furthermore, IBS-C and IBS-D shared one Megavirales species (Fig. 3C, Additional file 1: Table S4). This type of unique classification of Megavirales within IBS patients compares to HC also observed when we plotted the Megavirales relative abundances (Fig. 3D).

**Fig. 4:**
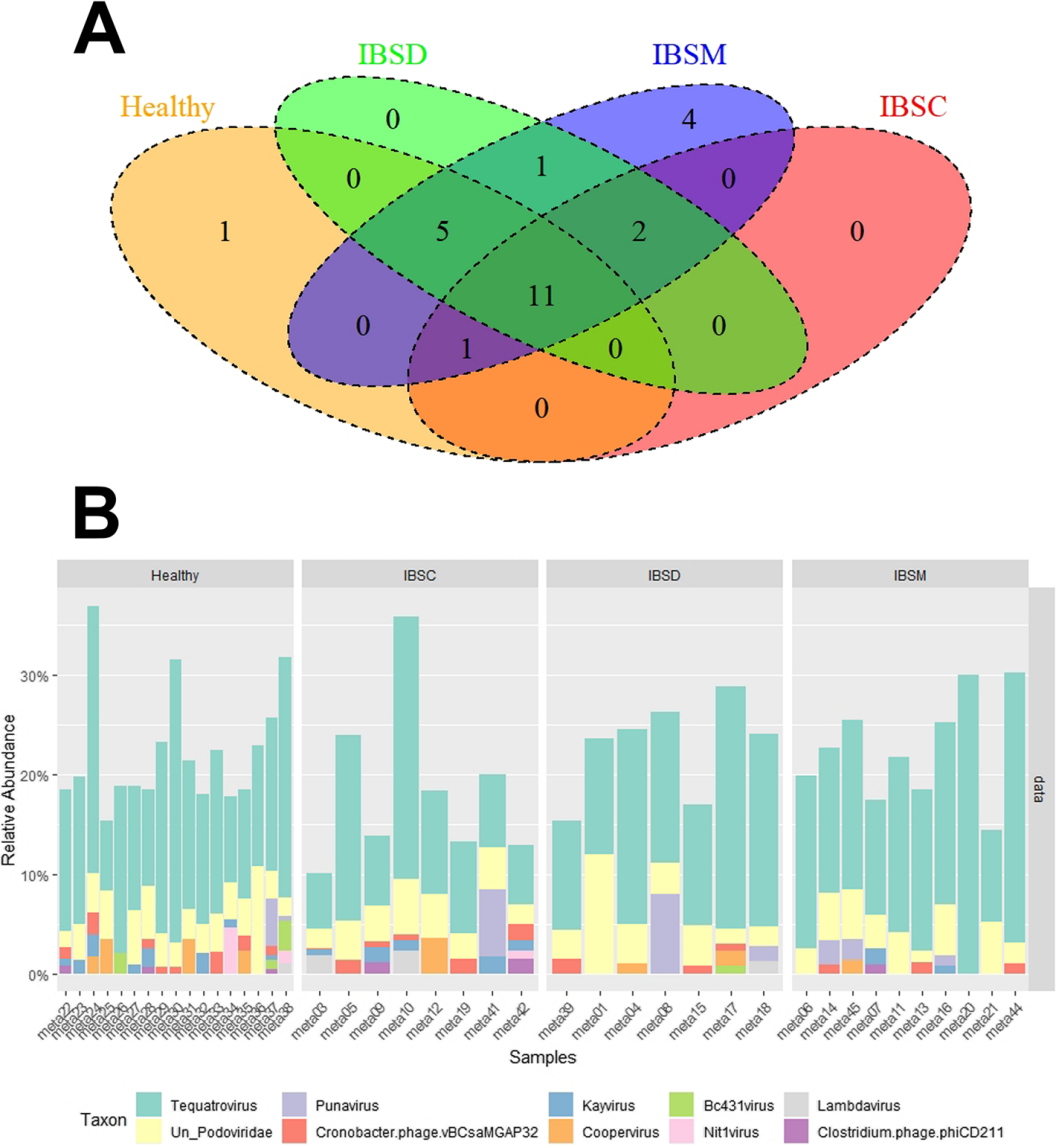
Caudovirales variation examined in association with healthy individuals and IBS patients. **a**: Venn diagram based on Caudovirales taxa in healthy control and different subtypes of IBS. *N* = the number of individuals in each group. **b**: The relative abundance of the 10 most abundant taxa of Caudovirales plotted for healthy control and different subtypes of IBS

On the other hand, no significant variation observed in the diversity of Caudovirales between patients with IBS or IBS subtypes and HC. However, the Venn diagram demonstrated that one Caudovirales species were uniquely presented in HC (Fig. 4A). Additionally, 11 species observed to be shared between HC and IBS subtypes (Fig. 4A). This result also indicated that all IBS subtypes shared in two Caudovirales species, but only IBS-M harbor four unique species of Caudovirales viruses (Fig. 4A, Additional file 1: Table S4). Plotting Caudovirales relative abundances indicated some variation in abundance of some taxa between HC and IBS patients and, in a general view, demonstrated also relatively higher diversity in HC compared to IBS patients, although — as we mentioned earlier — without significant support (Fig. 4B).

**Fig. 5:**
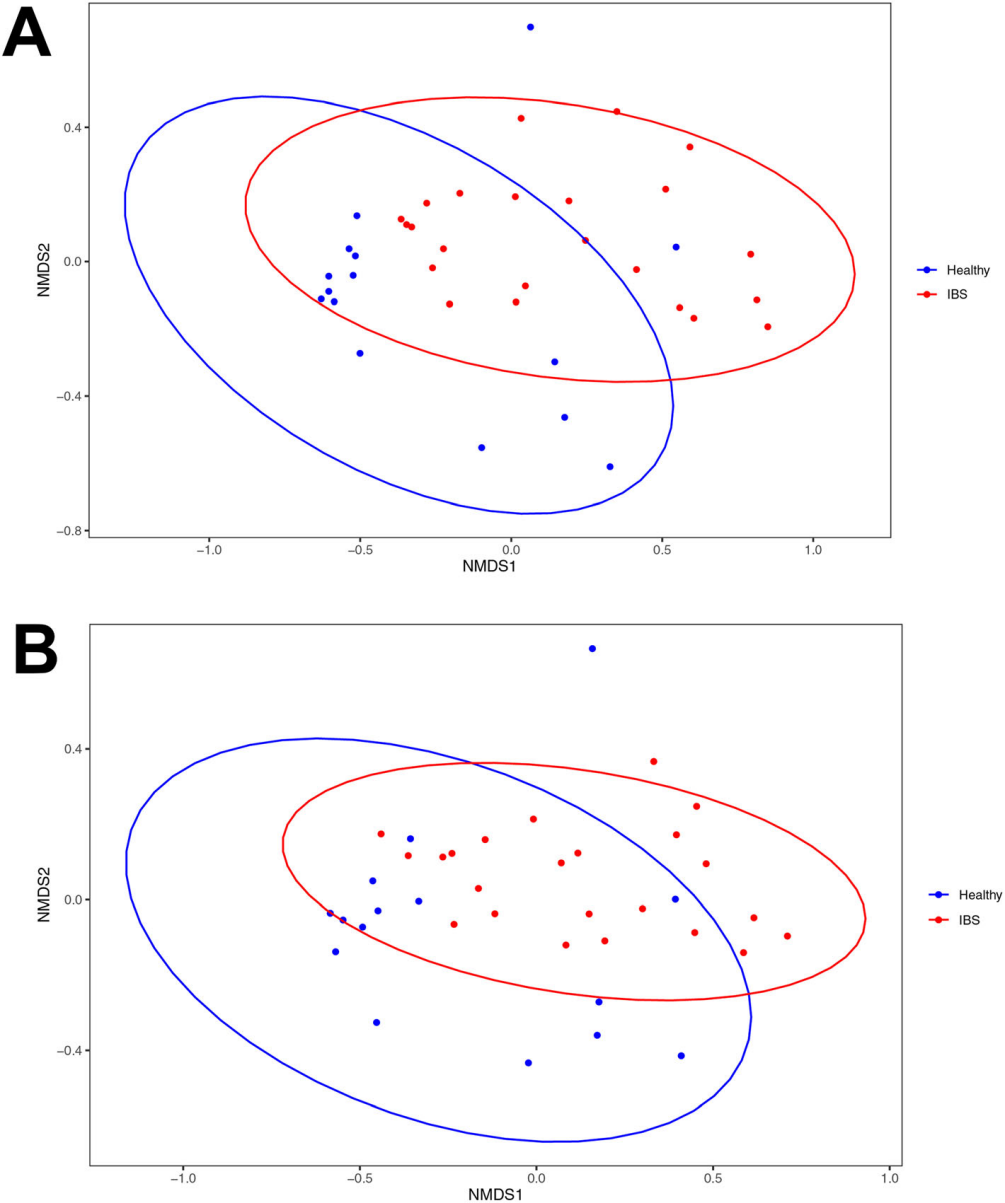
Non-metric multidimensional scaling (NMDS) plot of viral taxa based on OUT level derived from healthy controls and patients with IBS. Red dots within the red circle represent IBS patients and Blue dots showing the healthy individuals. **a**: The NMDS test based on OUT level of all viral sequences obtained from healthy individuals and IBS patients. **b**: The NMDS analysis based on OUT level isolated from Megavirals taxa assigned to healthy individuals and IBS patients

Non-metric multidimensional scaling (NMDS) showed no strong clustering between HC and IBS. However, some degrees of differentiation observed between these two groups (Fig. 5A), as Wilcoxon rank sum test also illustrated there are significant differences between HC and IBS of NMDS1 (*p* = 0.008 Wilcoxon test) and NMDS2 (*p* = 0.036 Wilcoxon test). The NMDS analysis for Megavirals also indicated some level of variation between HC and IBS (NMDS1 *p* = 0.003 Wilcoxon test; NMDS2 *p* = 0.031 Wilcoxon test, Fig.5B).

**Fig. 6:**
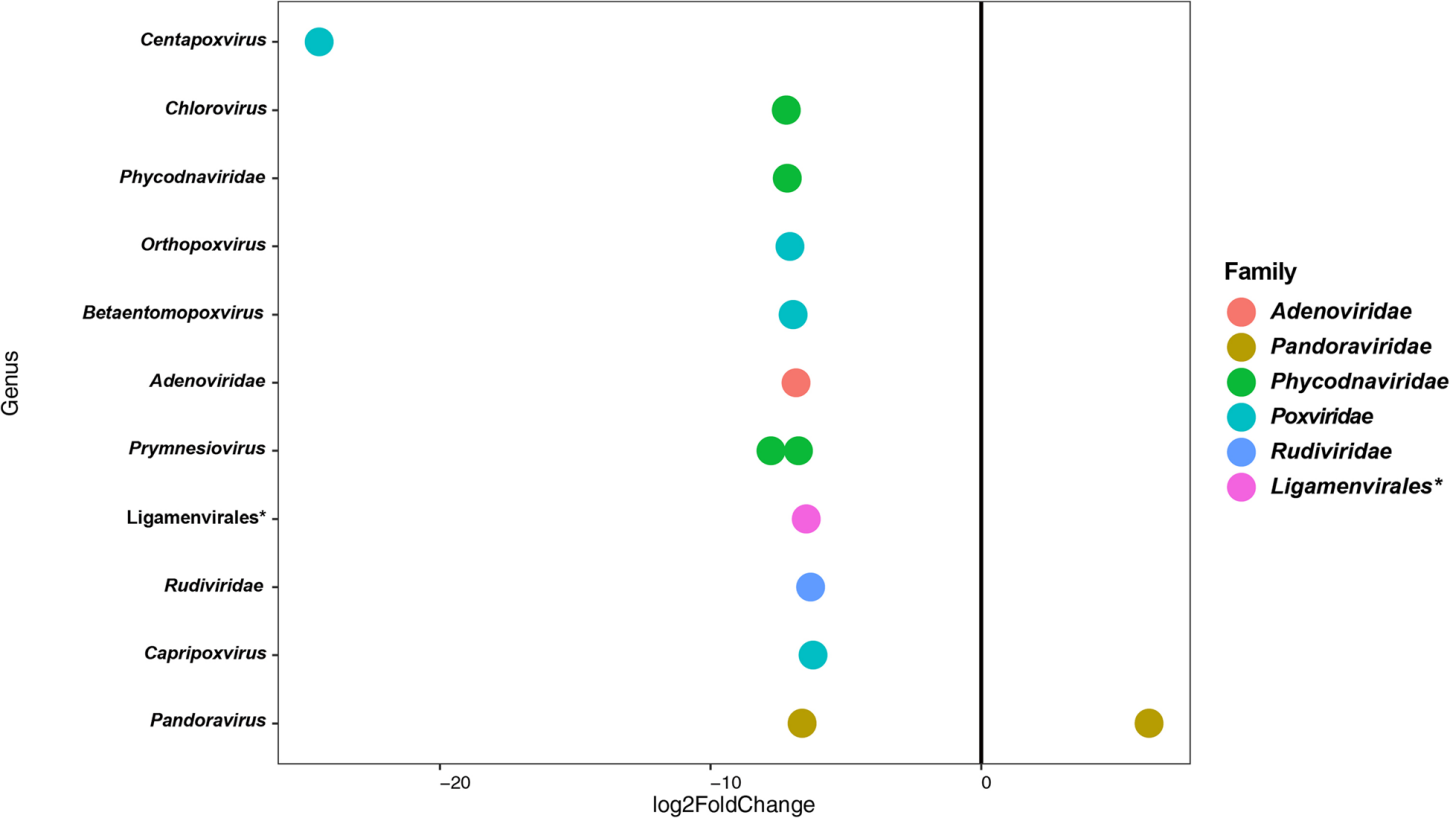
Pairwise comparison (DeSeq2 analysis). Variation in abundance OTUs (*p* < 0.05) between Healthy control and IBS patients. OTUs at the genus level (y-axis) and family level (colors). The Negative values of log2 Fold Change (x-axis) indicate higher relative abundance in IBS patients and positive values show for higher relative abundance in healthy individuals

The detailed OTU level base pairwise comparison between HC and IBS identified 13 OTUs abundance differences (DESeq analysis, *p* < 0.05, Fig. 6). *Pandoravirus salinus* from Megavirales order was the only species that significantly had higher abundance in IBS compared to HC, but four species from *Poxviridae*, four species from *Phycodnaviridae*, and one species from *Pandoraviridae* family showed lower abundance in IBS patients (Fig. 6). Additionally, species from *Adenoviridae* and *Rudiviridae* had lower abundance in IBS patients than healthy controls (Fig. 6, Additional file 1: Table S5).

## Discussion

One of the most challenging groups of gastrointestinal disorders are patients with IBS because the pathophysiology of the IBS is not well-defined and is also believed to be multifactorial, so the treatments of IBS are only moderately effective [1]. During the last decade, many studies have acknowledged the importance of altered gut microbiota composition in the onset of IBS [39–46]. Most of these studies assessed the bacterial composition of patients with a combination of IBS subtypes and reported an associated bacterial dysbiosis compared to healthy subjects [4]. In this study, we considered — for the first time — the possibility of changes in intestinal virome in association with IBS and assessed the viral community composition in a cohort of patients with IBS and compared the data to samples from healthy subjects.

The results of the current study demonstrated bacteriophages of the Caudovirales order were the most abundant viral taxa across the studied groups. Consistent to the current finding, bacteriophages have also been reported as one of the most abundant taxa from previous studies [35, 47, 48], but to our knowledge, there is no record of the potential alteration of the diversity or abundance of the specific taxa in association to IBS patients.

However, the small variation observed in the diversity and abundance of Caudovirales, which although showed no significant support, indicated some reduction in association with IBS patients, and could be related to two different scenarios. The first scenario is that Caudovirales could have a positive effect on disease prevention and treatment [49, 50], and so higher levels of taxa from Caudovirales would be expected to reside in the gut of healthy individuals. Therefore, the observed variation in the study might support the concept that Caudovirales have beneficial effects on human health.

On the other hand, it is well established that diet is a crucial driver of microbiome fluctuation [51–53] that is potentially mediates also the virome [54, 55]. Thus, individuals on different diets are likely to show more variation in gut virome compare to individuals with similar diets. Although we did not control for diet, we screened the participants for any supplementary diet or dietary restriction. However, a limitation to our study was the lack of completely controlled diets, leaving the potential that the Caudovirales variation we observed was caused by the subjects’ different eating patterns.

The eukaryotic gut viromes analysis, on the other hand, showed *Pandoravirus salinus* were highly abundant in IBS patients. As part of the human gut microbiome sequencing project, also many giant viruses were detected [56], yet their association with human health is indefinite [57]. In addition, we discovered that the *Herpesviridae* family was more abundant in patients with IBS compared to healthy subjects, even though the result showed no statistically significant support. We have not found any record about IBS patients to compare our findings with. However, in a previous study that compared the viral community composition between IBD patients and healthy individuals, *Herpesviridae* was also identified with a higher abundance in IBD patients [58]. It has been considered that *Herpesviridae* could be a significant regulator of homeostasis and inflammation of the intestine [59]. In this regard, *Herpesviridae* could continuously evade innate and adaptive immune systems [60, 61] and therefore may induce a chronic inflammatory response in IBS patients, a result in concordance to prior findings in patients with IBD and cancer [62, 63].

Other viral families, such as *Poxviridae*, *Phycodnaviridae*, *Pandoraviridae*, *Adenoviridae*, and *Rudiviridae* — that we detected to be less abundant in IBS patients — may be considered to be protective in the human host [28]. These viruses typically infect plants, amoebae, insects, and other animals, and their isolation from the human gut could be attributed to diet, although assessing the correlation between diet and fecal viruses of human needs further investigation [51].

Overall, our study suggests a new theory that the pathogenesis of IBS is potentially related to an alteration of gut virome composition, particularly eukaryotic virome composition. In addition to our results, which highlighted enteric viral dysbiosis between HC and patients with IBS, previously the pathogenesis of IBS has been related to the enteric bacterial dysbiosis [64, 65]. Therefore, these findings raise the question of whether alteration of whole comportment of the gut microbiome (including bacteria, viruses, fungus, etc.) or the interaction between different groups (such as bacteria and viruses) may be causing the symptomatic disease.

Of note, we provided viral data based on a small proportion of the general population and, even for those we were able to assess, many reads remain as unknown. Therefore, we can declare that we were able to only detect candidates related to the viruses available in the human gut, and we cannot dismiss the chance that the more relevant viruses to the pathophysiology of IBS could be hidden within the uncharacterized reads or unassessed proportion of the population. In addition, we only had a small number of patients from each of the IBS subgroups and we, therefore, compared the viral community composition between IBS patients as a combination of all subgroups and healthy individuals. Although we present the data for each IBS subgroups separately to provide an initial overview of how viral composition may be varied in IBS-subtypes, to assess the gut virome composition in association to each IBS subtype more precise study is required.

We believe this investigation is one of the first studies examining the viral community composition of microbiota in association to IBS, and future work should aim to assess the contribution of the viral alteration (both bacteriophages and eukaryotic viruses) to the etiology of IBS and its subtypes need to consider the limitation of the current study and increase sequencing power or choose bigger population group accordingly.

## Conclusions

Overall, the present study provides meaningful support for the importance of the viral community composition of gut microbiota in both human health and disease. The current results confirmed that *Myoviridae*, *Podoviridae*, and *Siphoviridae* were predominant families in the enteric viral community in both IBS and HC. However, we found that fecal viral diversity and abundance reduced in IBS patients compared to HC. *Pandoravirus inopinatum* significantly reduced in patients with IBS while *Pandoravirus salinus* noticeably had higher abundance in IBS subjects when compared to HC. Species from *Poxviridae*, *Phycodnaviridae*, *Adenoviridae*, and *Rudiviridae* also showed a higher abundance in HC compared to IBS patients. Even though the current findings are promising further studies are required to define the molecular mechanisms that gut viruses, especially eukaryotic viruses, cause gut inflammation and abnormalities of intestine motility, because their findings may help develop a new biomarker or drugs integrated into diagnosis and treatment of IBS. In addition, the current findings suggest a new hypothesis that the combination effect of whole component of gut microbiota (including bacteria, viruses, archaea, and fungus), or the interaction between them perhaps contribute to the onset of the disease, which should be considered by future studies research in IBS.

## Methods

### Study population and experimental design

We studied a total of 50 individuals, 30 of whom met the Rome IV criteria for IBS [66] and 20 healthy controls. Individuals were enrolled from the general population of Shiraz, Fars, Iran and Shiraz University of Medical Science Hospital outpatient clinics.

To determine eligibility all subjects were screened for inclusion criteria and considered eligible based also on their physical examination, medical history, and demographic characteristics.

The study criteria included subjects from 18 up to 50 years old of any sex or ethnicity. The healthy controls had no history of gastrointestinal (GI) symptoms in the past or present. All participants with a history of GI tract surgery (excluding appendectomy or cholecystectomy), a history of lactose malabsorption, diabetes, polyp, inflammatory bowel diseases (IBD), celiac disease (CD), any chronic disease including chronic kidney (CKD), chronic obstructive pulmonary (COPD) and collagen vascular disease, being pregnant or any other diagnosis that might clarify their irregular bowel symptoms were excluded from the experiments. In addition, subjects were excluded if they had any dietary restriction, or had received any supplementary diet; had been hospitalized; treated with antibiotics in the past 3 months prior to the experiment; regularly consumed aspirin or any other anti-inflammatory medicine such as nonsteroidal; or used immunosuppressive, synbiotics and/ or probiotics in the last 2 years prior to enrollment.

### Sample collection and DNA isolation

Fresh stool specimens were collected from all 50 participants on site during a study visit at Kavar (University cohort center) and Motahari outpatient clinic. Each fecal sample was immediately transferred on ice and were stored at − 80 °C. Then, total DNA was extracted from the samples using a NucleoSpin® Tissue DNA isolation kit (Genomic DNA from tissue, Macherey-Nagel, Germany), following the manufacture’s modified protocol for microorganism DNA from the stool.

### Library preparation and small whole-genome sequencing

The extracted genomic samples were prepared for sequencing using Nextera XT DNA Sample Prep Kit (Illumina Inc. San Diego, CA), following the manufacturer’s instructions. The protocols consist of several steps, including tagmentation, amplification, purification, normalization, and pooling libraries. Briefly, 1 ng of template was used for the tagmentation of the samples, followed by PCR amplification with a unique combination of barcode primers. Next, the PCR products were purified using Agencourt AMpure XP beads (Beckman Coulter). The purified products were then normalized, and equal volumes of normalized libraries were pooled together. The pooled libraries sequenced on the Illumina Nextseq platform with a 2 × 150 paired-end read length at the Faghihi Medical Genetics center (FMG; Shiraz, Fars, Iran) following standard protocols.

### Bioinformatics and taxonomic assignment

The qualities of raw reads files from small whole-genome sequencing were assessed using FASTQ (http:// www. Bioinformatics. Babraham. ac.uk/projects/fastqc/). The raw fastq files were filtered by low-quality bases (Phred score ≥ 20) and no acceptance of ambiguous bases. Adaptors and host-specific sequences also were trimmed using CLC Genomic Workbench (CLC bio) and filtered reads longer than 50 bp were subjected to the following analysis.

The trimmed sequences were clustered at a 97% similarity level using CLC microbial genomic module v 3.0, for obtaining a unique sequences dataset. The sequences were then blasted against the viral RefSeq database. Finally, for taxonomic assignment, the lowest-common ancestor algorithm was applied as implemented in MEGAN V6.11.6 [67]. The settings used for the analysis were included: Min Support = 1, Min Score = 40.0, Max Expected = 0.01, Top Percent = 10.0, Min Complexity = 0.4 with the Min-Complexity Filter turned on. 

### De novo contig assembly

For assembling the contigs, the trimmed contigs were introduced to IDBA_DA assembler (v 1.1.0) with minimum and maximum kmer lengths of 20 and 120, respectively [68]. All resulted contigs larger than 300 nucleotides were blasted against a viral genome, including 7696 viral sequences available in NCBI as on February 17, 2018.

### Statistical analysis

In order to do statistical analysis, absolute read counts of viromes were exported from MEGAN and introduced into the R program. Phyloseq package/R [69] was used for general visualization and beta diversity analysis of the sequence data. We used taxa with more than 25% prevalence in the subjects for subsequent analysis. Data were normalized using cumulative sum scaling method (CSS) of metagenomSeq R/Bioconductor package in R-Studio [70–72]. The vegan package/R was used to calculate richness and diversity [73]. Non-parametric Wilcoxon rank-sum test was used to compare diversity between HC and IBS and kruskal-wallis test was used for comparing HC and all IBS subtypes as our data were not normal according to Shapiro-Wilk test. The cor and cor.test function in the R project was used to calculated Spearman correlation and test its significance, respectively. In order to visualize microbial abundance differences between HC and IBS we used Non-metric multidimensional scaling (NMDS) based on Bray–Curtis dissimilarity on normalized data (vegan package). We then used Wilcoxon rank-sum test to determine if there was any significant difference between HC and IBS samples of first and second NMDS [64]. Negative Binomial log-linear model of DESeq2 package/R was used to identify different species abundance between HC and IBS patients [74, 75]. Correlation plots and heat maps were generated using the corrplot R package [76] and the pheatmap R package [77], respectively. The Benjamini-Hochberg procedure which controls the false discovery rate (FDR) was used to adjust *p*-value for multiple comparisons.

## Supplementary information

**Additional file 1: Table S1.** The demographic characteristics and medical history of the volunteers. **Table S2.** Detailed information about the developed sequences. **Table S3.** The result of correlation between different viral orders tested for each IBS subtypes and HC separately. **Table S4.** The viral species assigned to the sequences obtained from healthy control (HC) and different subtypes of IBS patients. Present = 1 and Absent = 0. **Table S5.** Variation in abundance of viral taxa derived from healthy individuals and patients with IBS. The significant variation in abundance of viral taxa in association with IBS determined using DESeq2 analysis *P* < 0.05.

**Additional file 2: Figure S1.** The relative abundance of sequences assigned to the viral orders in association with healthy control and IBS patients. **Figure S2.** The relative abundance of sequences assigned to the viral family in association with healthy control and IBS patients.

## Data Availability

The sequences can be accessed after 2021 01 25 at MG-RAST (http://metagenomics.anl.gov/) server under the project name IBS-Ansari(MG-Rast ID mgs799371– mgs799344). Until then, the sequences are available from the corresponding author upon reasonable request.

## Abbreviations

IBS: Irritable bowel syndrome
IBS-C: Predominant constipation
IBS-D: Diarrhea-predominant
IBS-M: Patients with mixed-bowel symptoms
HC: Healthy controls
IBD: Inflammatory bowel disease
GI: Gastrointestinal
CD: Celiac disease
CKD: Chronic kidney
COPD: Chronic obstructive pulmonary
FMG: Faghihi Medical Genetics center
PCR: Polymerase chain reaction
