# Supplementary material for: Viral metagenomic analysis of fecal samples reveals an enteric virome signature in irritable bowel syndrome
Source: BMC Microbiol. 2020 May 19;20:123. doi: 10.1186/s12866-020-01817-4 (PMC7236503; doi:10.1186/s12866-020-01817-4)
Supplement: Supplementary file 2 — Additional file 2: Figure S1. The relative abundance of sequences assigned to the viral orders in association with healthy control and IBS patients. Figure S2. The relative abundance of sequences assigned to the viral family in association with healthy control and IBS patients. [file 12866_2020_1817_MOESM2_ESM.pdf]

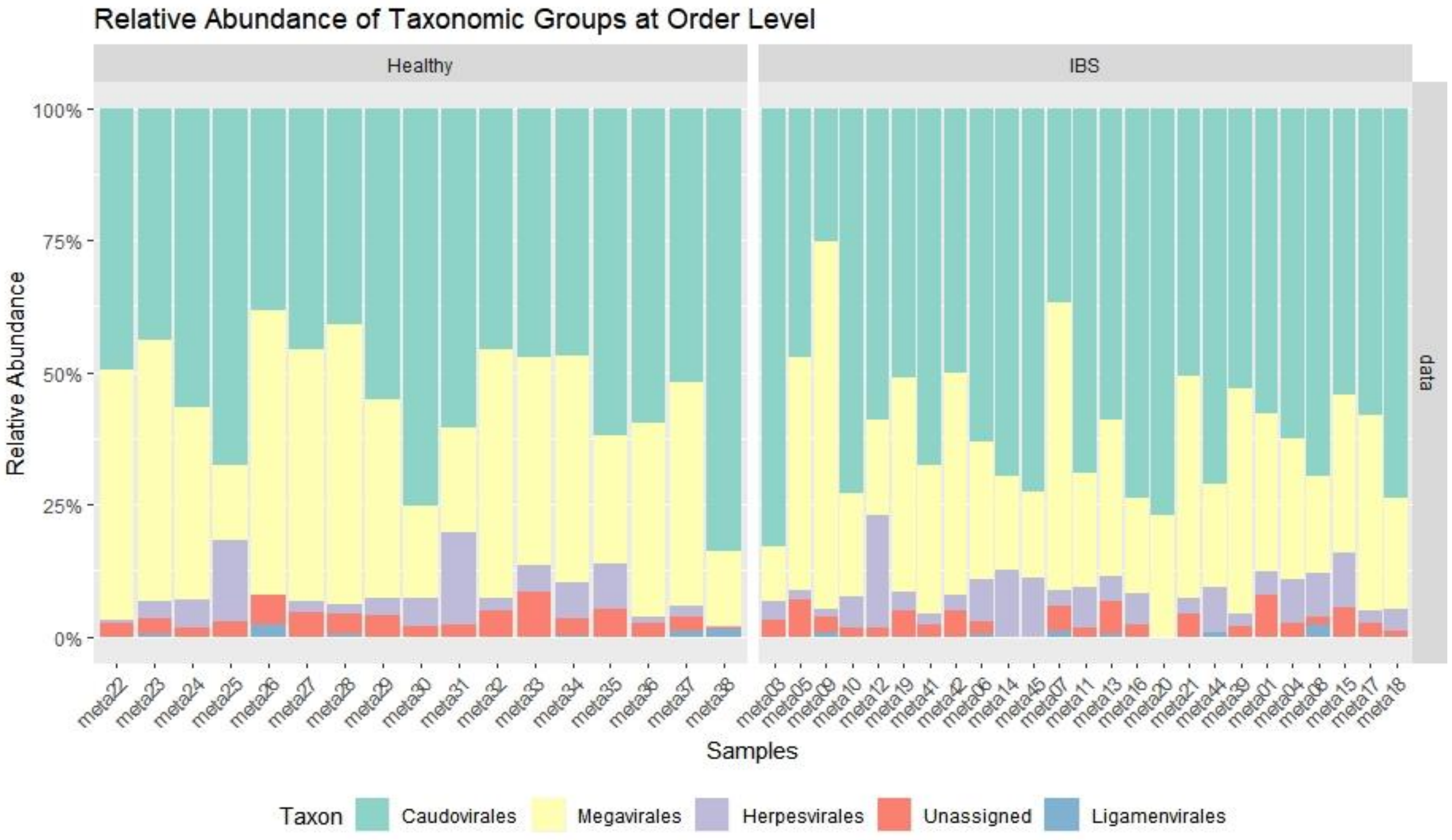

Additional file 2: **Fig. S1.** Relative abundance of sequences assigned to the viral orders in association with healthy control and IBS patients.

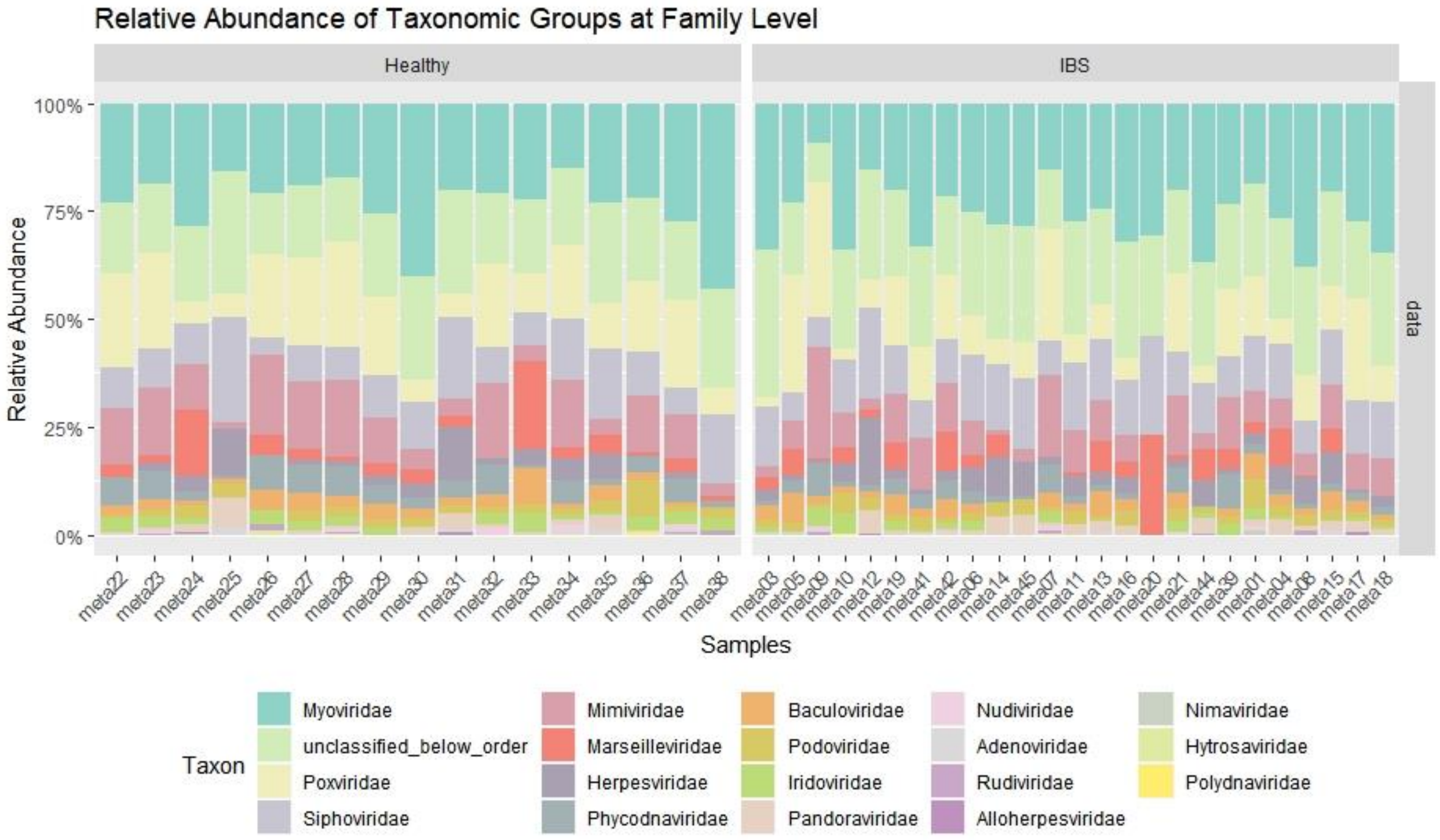

Additional file 2: **Fig. S2.** Relative abundance of sequences assigned to the viral family in association with healthy control and IBS patients.
